# Human μ Opioid Receptor Models with Evaluation of the Accuracy Using the Crystal Structure of the Murine μ Opioid Receptor

**DOI:** 10.4172/2155-6148.1000218

**Published:** 2012-07-02

**Authors:** Jose Manuel Perez-Aguilar, Jeffery G. Saven, Renyu Liu

**Affiliations:** 1 Department of Chemistry, University of Pennsylvania, Philadelphia, 19104, USA; 2 Department of Anesthesiology and Critical Care, Hospital of University of Pennsylvania, Philadelphia, 19104, USA

**Keywords:** Human μ opioid receptor, Murine μ opioid receptor, G-protein-coupled receptor (GPCR), Homology modeling

## Abstract

Models of the human μ opioid receptor were constructed using available G-protein-coupled receptor (GPCR) structures using homology (comparative) modeling techniques. The recent publication of a high-resolution crystal structure of a construct based on the murine μ opioid receptor offers a unique opportunity to evaluate the reliability of the homology models and test the relevance of introducing more templates (known structures) to increase the accuracy of the comparative models. In the first model two templates were used: the β_2_ adrenergic and bovine rhodopsin receptors. For the second model, four templates were utilized: the β_2_ adrenergic, bovine rhodopsin, β_1_ adrenergic, and A_2A_ adenosine receptors. Including additional templates improved the accuracy of structural motifs and other features of the model when the same sequence alignment was used. The predicted structures were especially relevant in the case of important receptor regions such as the DRY motif, which has been associated with receptor activation. Additionally, this study showed that receptor sequence similarity is crucial in homology modeling, as indicated in the case of the highly diverse EC2 loop. This study demonstrates the reliability of the homology modeling technique in the case of the μ opioid receptor, a member of the rhodopsin-like family class of GPCRs. The addition of more templates improved the accuracy of the model. The findings regarding the modeling has significant implication to other GPCRs where the crystal structure is still unknown and suggest that homology modeling techniques can provide high quality structural models for interpreting experimental findings and formulating structurally based hypotheses regarding the activity of these important receptors.

## Introduction

Opioid receptors are part of the largest family of integral transmembrane proteins coded by the human genome, the G-protein-coupled receptors (GPCRs) [1]. GPCRs mediate most transmembrane signal transduction, usually in response to hormones, neurotransmitters and environmental stimulants. Each GPCR comprises an extracellular N terminus, seven-transmembrane (7TM) helical segments separated by alternating intracellular and extracellular loop regions, and an intracellular C terminus [1–3]. Opioid receptors are part of the largest family of GPCRs, family A or rhodopsin-like GPCRs [4]. Other family A members include the receptors for epinephrine, dopamine, serotonin, and adenosine [5]. The μ opioid receptor is the primary receptor in the brain for endogenous opioid neuropeptides as well as exogenously administrated opioid compounds [6–8]. Potent drugs such as morphine, heroin, fentanyl and methadone induce their pharmacological effects through the activation of this receptor [9].

Extensive computational comparative modeling of the μ opioid receptor was used to suggest structural details of this important signal transduction protein [10–15] before the crystal structure of the murine μ opioid receptor was revealed [16]. The μ opioid receptor has been heavily modeled using the few receptor structures available at the time due to its importance related to addiction and pain control and reward pathways [6,7,12,14]. More recently, the addition of several GPCR structures in recent years opens the potential opportunity for higher quality modeled structures. Within the past few years, our group has constructed different versions of homology models of human μ opioid receptor (hMOP-R) based on the available structural information at that time. The recent publication of a high-resolution crystal structure of murine μ opioid receptor solution offers a unique opportunity to evaluate the reliability of the modeling of GPCRs of this family using other GPCR structures and to test the relevance of introducing more templates to increase the accuracy of the comparative models.

## Methods

Two different homology models were constructed in our group before the crystal structure of murine μ opioid receptor was disclosed: (i) The first model, named as 2T-hMOP-R, used the X-ray crystallographic structures of human β_2_ adrenergic receptor at 2.4 Å resolution (PDB accession code: 2RH1) [17] and bovine rhodopsin at 2.2 Å resolution (PDB accession code: 1U19) [18] as templates. (ii) the second model, named as 4T-hMOP-R, used the X-ray crystallographic structures of turkey β_1_ adrenergic receptor at 2.7 Å resolution (PDB accession code: 2VT4) [19] and human A2A adenosine receptor at 2.6 Å resolution (PDB accession code: 3EML) [20] in addition to the above mentioned 2 templates.

Given the importance of sequence alignments in the comparative modeling procedure [21–23], several different programs and substitution matrices were considered. The sequences of hMOP-R, human β_2_ adrenergic receptor, bovine rhodopsin, turkey β_1_ adrenergic receptor and human A_2A_ adenosine receptor were obtained from the UniProt Knowledgebase UniProtKB server with the accession numbers P35372, P07550, P02699, P07700, and P29274, respectively [24]. *BLASTp* [25], *SIM* [26], *ClustalW* [27], and *Phyre* [28] were used to align the sequences of hMOP-R and human β_2_ adrenergic receptor. In the case of *BLASTp*, three members of the “Blosum” substitution matrix family [29] (Blosum62, Blosum45 and Blosum80) and one member of the “PAM” substitution matrix family [30] (PAM70) were used. For the case of the alignment tool SIM, Blosum62 and Blosum30 were used. For *ClustalW* just the Blosum30 matrix was used. The protein structure prediction server *Phyre*, was also utilized. Standard penalty gaps were applied in all the cases [25–27]. A similar procedure was carried out in the case of hMOP-R and bovine rhodopsin, hMOP-R and β_1_ adrenergic receptor, and hMOP-R and A_2A_ adenosine receptor.

**Figure 1:**
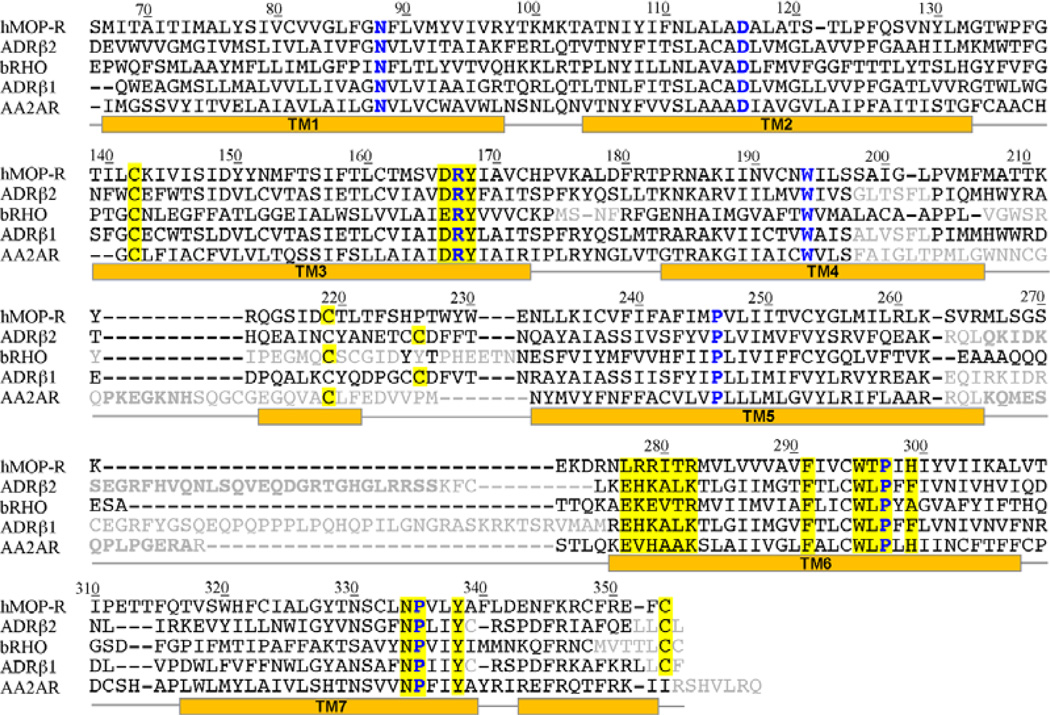
Sequence alignment used in the creation of the models of the human μ opioid receptor, hMOP-R. The templates are: human β_2_ adrenergic receptor (ADRβ_2_), bovine rhodopsin (bRHO), turkey β_1_ adrenergic receptor (ADRβ_1_) and human A_2A_ adenosine receptor (AA2AR). The residues of the N- and C- terminus are excluded (residue 1 to 65 and residues 354 to 400, respectively). Also, the residues excluded from the comparative modeling are colored in gray. The most conserved residues at each of the transmembrane helices are depicted in blue. The secondary structure of the β_2_ adrenergic receptor based on STRIDE [32], is shown below the sequences. Residue numbering of hMOP-R is shown. Highly conserved motifs in the rhodopsin-like GPCR family are highlighted in yellow.

A final sequence alignment was obtained for each pair of proteins and modifications were performed to maintain highly conserved fingerprint residues of the rhodopsin-like GPCR family [31]. Among these are: the *disulfide bond* between TM3 and the second extracellular loop (EC2), the “DRY” motif in TM3, the *XBBXXB* motif in the third intracellular loop (IC3) (where B represents a basic amino acid and X represents a non-basic residue, LRRITR in the case of hMOP-R), the *FXXXWXPX*[*F*] motif in TM6 (*FIVCWTPIH* in the case of hMOP-R), the *NPXXY* motif in TM7 (*NPVLY* in hMOP-R), and the C-terminal cys palmitoylation site [31]. The final multiple sequence alignment is presented in figure 1.

Using the sequence alignments and the two- and four-template structure sets described above, one hundred models of the human μ opioid receptor models (from residue 65 to 353) in each case were generated using Modeller 9v2 with the refinement optimization level adjusted to *slow* [32,33]. The side chains from the resulting models of the four-template and two-template ensembles, were minimized using NAMD2 [34] and the CHARMM22 force field [35]. Hydrogen atoms were added and minimization was performed by the conjugate-gradient method until the total energy remains constant (change in energy less than 1.0 kcal/mol). Models with the lowest energy were selected and characterized using Molprobity [36] to confirm that no steric clashes or unusual conformations of the backbone and side chains were present. Herein, the selected structures from the four- and two-template sets are denominated 4T-hMOP-R and 2T-hMOP-R, respectively. The secondary structures of the models were assigned with STRIDE [37], and the locations and lengths of the TM helices were the same for both 4T-hMOP-R and 2T-hMOP-R. The X-ray crystallographic structure murine μ opioid receptor (PDB accession code: 4DKL) was used to evaluate these models. Renderings of molecular structures for comparison were generated using PyMOL (http://www.pymol.org/, Version 1.3, Schrödinger, LLC).

## Results and Discussion

### Comparison between models 4T-hMOP-R and 2T-hMOP-R

**Figure 2:**
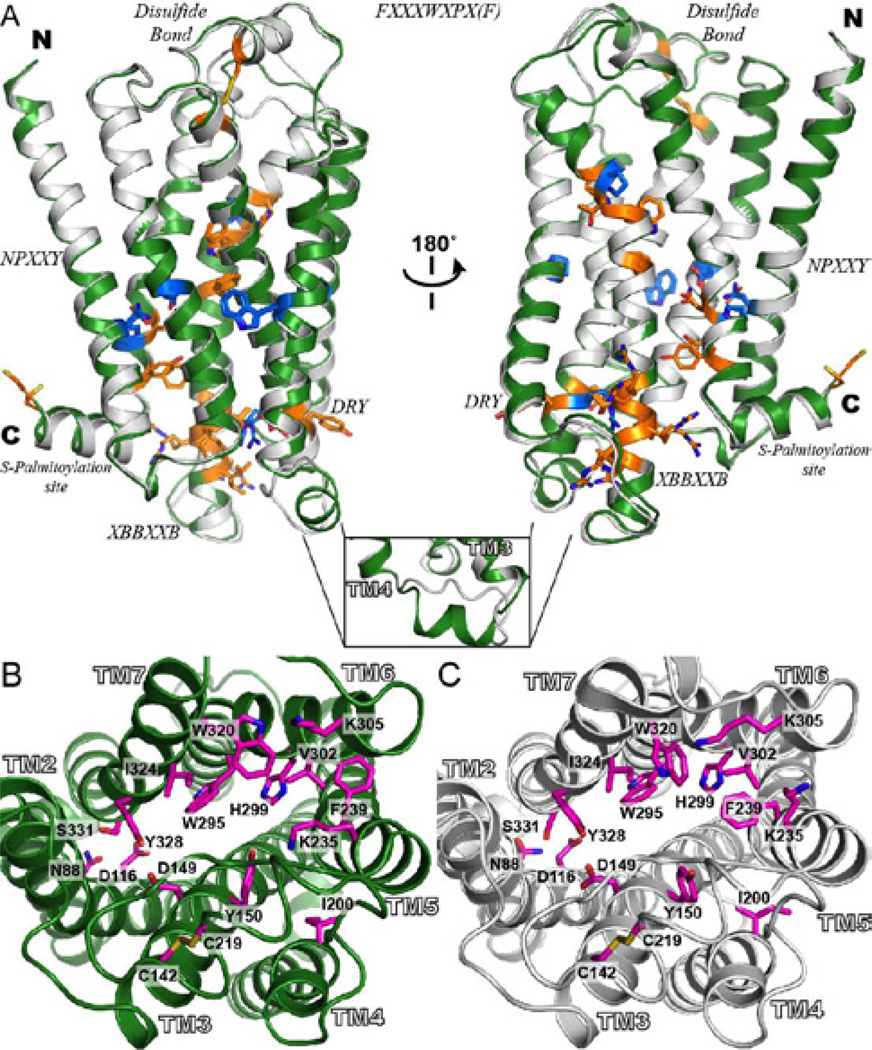
Alignment and comparison of representative models of hMOP-R. (A) the representative models from the four-template ensemble (4T-hMOPR) and two-template ensemble (2T-hMOP-R) are color in green and white, respectively. The most conserved residues at each of the transmembrane helices are depicted as blue sticks. The highly conserved motifs in the rhodopsin-like GPCR family are depicted as orange sticks; disulfide bond between TM3 and EC2, DRY in TM3, XBBXXB in IC3 (where B represents a basic amino acid and X represents a non-basic residue), FXXXWXPX[F] in TM6, the NPXXY in TM7 and the C-terminal cys palmitoylation site. The inset at the bottom shows one of the most significant differences between the two models. The IC2 loop that connects helices TM3 and TM4 in the 4T-hMOPR model, forms a helical motif inherit from the β1 adrenergic receptor and the A2A adenosine receptor templates. (B) Top views of the binding site for the 4T-hMOP-R and 2T-hMOP-R models with a set of the relevant residues involved in ligand interaction based on mutagenesis studies. Side chain residues are colored in magenta in both cases. This view of the hMOP-R from the extracellular side of the membrane shows the counterclockwise arrangement of the TM helices.

Two different views of the model of hMOP-R are depicted in Figure 2a. In general, 4T-hMOP-R and 2T-hMOP-R are similar with a backbone rmsd of 1.30 Å. One of the most significant differences between the models is a helical segment in IC2 that is specific to the β_1_ adrenergic receptor and A_2A_ adenosine receptor; it is absent in 2T-hMOP-R (see inset of Figure 2a). This loop connects helices TM3 and TM4 and is close to the important and highly conserved *DRY* motif. In general, both structures could be used to interpret different experimental studies associated with ligand binding properties of the μ opioid receptor (see Figure 2b).

### Comparison of the constructed models with the crystal structure of mouse μ opioid receptor

The human (uniprot accession number P35372) and mouse (uniprot accession number P42866) μ opioid receptors has a sequence identity of 94% if the entire receptor sequences are considered and a sequence identity of 99% for the structure solved in the crystal structure. The sequence identity between human and mouse μ opioid receptor suggests that both proteins likely share a very similar structure.

**Figure 3:**
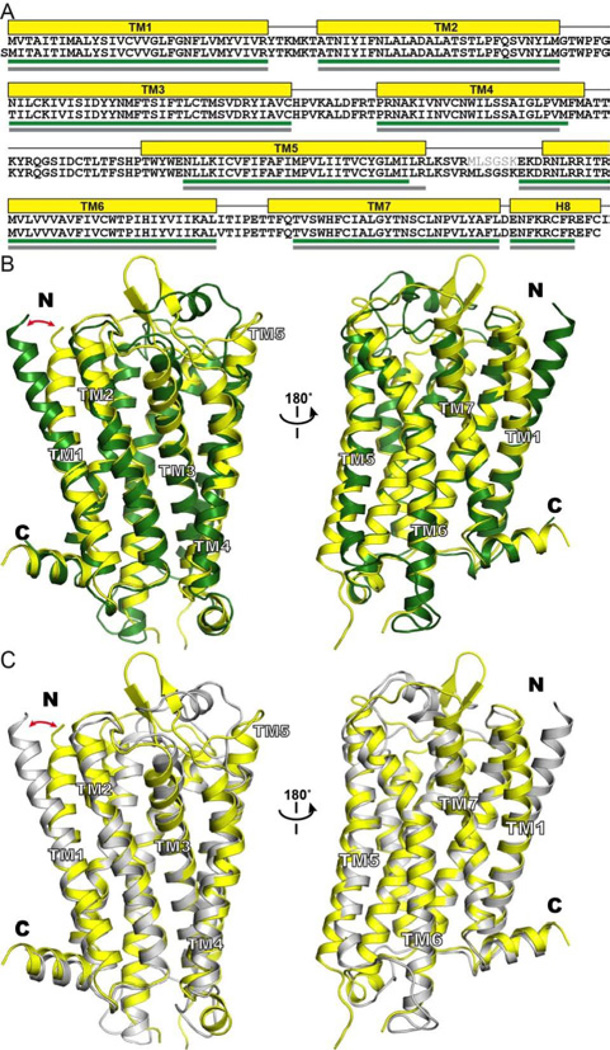
Comparison of the structure of mouse μ opioid receptor with the models of the human μ opioid receptor 4T-hMOP-R and 2T-hMOP-R. (A) Sequence alignment that displays the length of the TM helices in the crystal structure (top sequence) and the 4T-hMOP-R and 2T-hMOP-R models (bottom sequence). The helical segments are depicted as yellow boxes for the crystal structure and as green and gray lines for 4T-hMOP-R and 2T-hMOPR, respectively. (B) Crystal structure (yellow) and the 4T-hMOP-R model (green) are superimposed. (C) Crystal structure (yellow) and the 2T-hMOP-R model (gray) are superimposed. The red arrow indicates the different position of the extracellular half of the TM1 helix.

The representative models from the comparative modeling procedure were compared with the crystal structure of murine μ opioid receptor (PDB accession code: 4DKL). The root-mean-square deviation (rmsd) of the Cα atoms located in the TM helices (Figure 3A) between the crystal structure and 4T-hMOP-R and 2T-hMOP-R is 2.67 Å and 2.60 Å, respectively. Superposition of the structures is shown in figure 3. As seen from the rmsd values, both modeled structures are, in general, very similar to the crystal structure. Interestingly, one of the main differences comes from the extracellular portion of TM1. In the crystal structure, this segment of TM1 presents a position that is closer (~ 10 Å) to the rest of the helical bundle (Figure 3B and 3C). This relative position is not seen in any of the templates and thus, not present in the models. Interestingly and despite the sequence identity, the recent structure of a closely related receptor, the human κ opioid receptor [16] presents the same segment of TM1 with an outward displacement similar to the templates (and the models) presented here. This displacement has been suggested to reflect difference in crystallization conditions or crystal packing [19–38].

### The extracellular loop (EC2)

**Figure 4:**
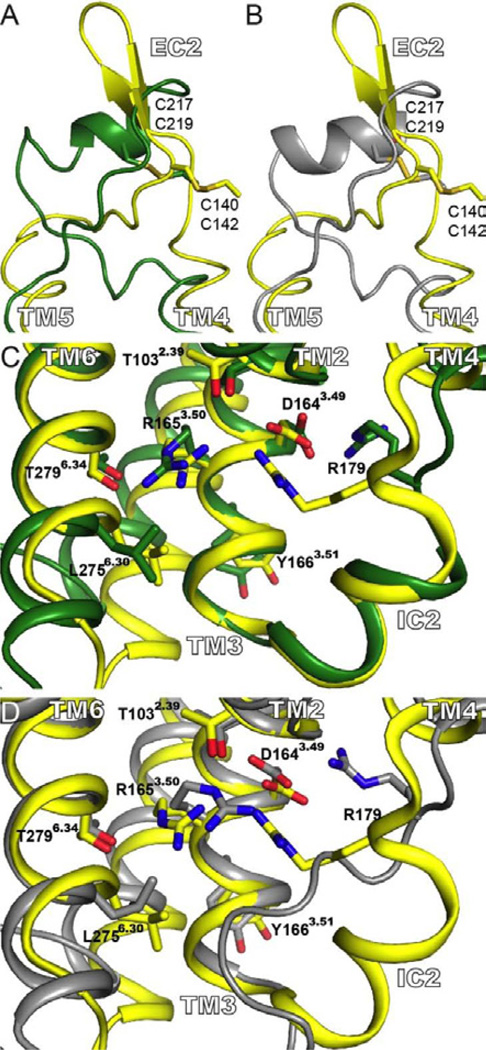
Structure of the EC2 and IC2. (A) and (B) Superposition of the EC2 for the crystal structure (yellow) and the 4T-hMOP-R (green) and 2T-hMOP-R (gray) models, respectively. The conserved disulfide bond between C140 in TM3 and C217 in EC2 is displayed. The numbers below correspond to the residue number for the equivalent residues in the human μ opioid receptor (C219 and C142). (C) and (D) display views from the intracellular milieu, particularly interactions around the DRY motif. In (C) a comparison of the crystal structure (yellow) with 4T-hMOP-R (green) is shown. In (D) comparison of the crystal structure (yellow) with 2T-hMOP-R (gray) is shown. Relevant side chains are depicted.

An interesting case is the structure adopted by the second extracellular loop (EC2) that, when compared with TM region, presents a larger sequence diversity among the μ receptor and the template proteins. In the models the structure of EC2 was modeled mainly using the information from the β_2_ adrenergic receptor in 2T-hMOP-R and β_2_ and β_1_ adrenergic receptors in 4T-hMOP-R. In both cases, EC2 forms a short helix, partially inherited from the adrenergic receptors. In the crystal structure of murine μ opioid receptor the EC2 loop forms a β-sheet structure. Interestingly, the positions of the cysteine residues that form the highly conserved disulfide bond were predicted correctly in both models figure 4A and 4B.

### The conserved DRY motif

Another important feature is the set of interactions around the conserved DRY motif (Figure 4C and 4D). In bovine rhodopsin, the highly conserved residue R135^3.50^ is forming a salt-bridge with E2476.30. This interaction, sometimes denominated “ionic lock”, is not present in the crystal structure of the μ opioid receptor (also this interaction is not seen in the other three templates utilized in this study). In the case of the crystal structure of the μ opioid receptor, the equivalent position, R165^3.50^, is interacting with the side chain of T279^6.34^. This polar interaction is correctly predicted in both, 4T-hMOP-R and 2T-hMOP-R structures, (Figures 4C and 4D). In general terms, most of the residues around the DRY motif, as well as the interactions, are properly predicted in both representative models. The exception is the conformation of the long side chain of residue R179 located in the IC2 loop. The helical structure of this loop in 4T-hMOP-R is in good agreement with the structure seeing in the crystal structure, figure 4C. Because neither bovine rhodopsin nor the β_2_ adrenergic receptor presents helical content in this loop, the structure of IC2 in 2T-hMOP-R does not reproduce correctly the topology seen in the crystal structure. Despite the correct prediction of the helical content of IC2 in 4T-hMOP-R, the conformation of the long side chain of R179 is not properly modeled, even though the interaction of R179 with D164^3.49^ is correctly predicted in both 4T-hMOP-R 2T-hMOP-R and (Figure 4D).

### The binding pocket

**Figure 5:**
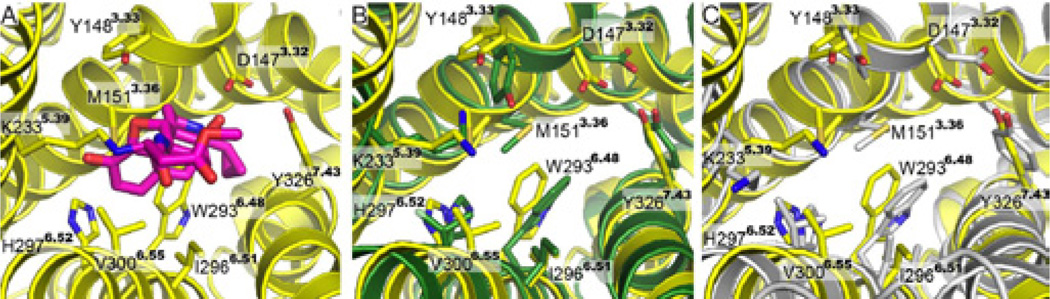
Binding pocket comparison. (A) The binding pocket of the crystal structure of mouse μ opioid receptor with the structure of β-FNA (magenta) is displayed. The nine residues that directly interact with β-FNA are indicated. (B) and (C) Comparison of the nine residues in the binding pocket of the crystal structure (yellow) and the 4T-hMOP-R (green) and 2T-hMOP-R (gray) models, respectively.

The analysis of the binding pocket in the crystal structure of the μ opioid receptor shows important features that are conserved in the model structures. Both, 4T-hMOP-R and 2T-hMOP-R correctly orient the side chain of K233^5.39^ toward the binding pocket where it could covalently bind β-FNA, as observed in the crystal structure. The nine positions that directly interact with β-FNA in the crystal structure (D147^3.32^, Y148^3.33^, M151^3.36^, K233^5.39^, W293^6.48^, I296^6.51^, H297^6.52^, V300^6.55^, and Y326^7.43^) are displayed in figure 5A. These residues from the crystal structure are compared with the equivalent residues in 4T-hMOP-R and 2T-hMOP-R (Figures 5B and C). In general, the orientation of the side chains of these nine residues was correctly modeled with rmsd values with the side chains atom of the crystal structure of 1.4 Å and 1.6 Å for the 4T-hMOP-R and 2T-hMOP-R, respectively, even though no ligand was present in creating the models.

In conclusion, using the newly available crystal structure the murine μ opioid receptor, we demonstrated the relative accuracy of the homology modeling for μ opioid receptor. The addition of more templates improved the accuracy of the model. This was especially relevant in the case of important receptor regions such as the DRY motif, which has been related with receptor activation, and the ligand binding pocket. Additionally, this study shows that some degree of receptor sequence similarity is useful in homology modeling: in the case of the loop EC2 where little consensus in about the alignment was observed, a β-sheet rather than α-helical structure was observed in the crystal structure. The findings have significant implication for the construction of model structures of GPCRs, particularly those of the same family, where crystal structures are still unavailable. Such models can guide the interpretation of experimental findings, the creation of structure-based models for receptor activation, and the formulation of hypotheses regarding these important receptors.
